# 4483 Activity and Abundance of Mucus-degrading Microbes in Inflammatory Bowel Disease

**DOI:** 10.1017/cts.2020.288

**Published:** 2020-07-29

**Authors:** Matthew Ostrowski, Eric C. Martens

**Affiliations:** 1 University of Michigan School of Medicine; 2 University of Michigan

## Abstract

OBJECTIVES/GOALS: This study seeks to culture and characterize mucus-degrading microbes from the microbiome of inflammatory bowel disease (IBD) patients. METHODS/STUDY POPULATION: Stool will be collected from IBD patients and healthy first-degree relatives, then enriched for mucin-degrading microbes through growth on porcine rectal mucin. Dilution plating in both liquid and solid culture formats will be employed to isolate strains capable of growth on mucin. Cultures that are positive for mucin degradation will be identified with 16S rRNA sequencing; unique isolates will be genome sequenced and transcriptionally profiled on simple monosaccharides and mucin in order to identify putative mucin-degrading genes. The abundance of novel enzymes, pathways, and microbes will be compared in healthy and IBD patient populations using existing datasets in the literature. RESULTS/ANTICIPATED RESULTS: We expect to isolate previously uncultured mucin-degrading microbes, which will likely include new strains and possibly new species of bacteria. Through the transcriptomic characterization of mucin-degrading pathways, we will expand the lexicon of known mucin-degrading enzymes and pathways used by bacteria in the human colon. We expect mucin-degrading microbes to be more abundant and active in IBD patients compared to healthy controls. DISCUSSION/SIGNIFICANCE OF IMPACT: There is no cure for IBD and treatment relies heavily on suppressing a patient’s immune system. This research seeks to understand the contribution of the gut microbiota in the pathogenesis of IBD, which may lead to future therapeutic targets.

